# Prognostic factors of primary neuroendocrine breast cancer: A population‐based study

**DOI:** 10.1002/cam4.4557

**Published:** 2022-05-02

**Authors:** Shu‐tao Ma, Ding‐yuan Wang, Yi‐bing Liu, Hui‐jing Tan, Yue‐yue Ge, Yihebali Chi, Bai‐lin Zhang

**Affiliations:** ^1^ Department of Oncology, National Cancer Center/National Clinical Research Center for Cancer/Cancer Hospital Chinese Academy of Medical Sciences and Peking Union Medical College Beijing China; ^2^ Department of Breast Surgery, National Cancer Center/National Clinical Research Center for Cancer/Cancer Hospital Chinese Academy of Medical Sciences and Peking Union Medical College Beijing China; ^3^ Third Clinical Medical College Jilin University Changchun China; ^4^ Department of Geriatric Medicine, National Center for Clinical Laboratories, Beijing Hospital National Center of Gerontology; Chinese Academy of Medical Sciences Beijing China

**Keywords:** breast cancer, endocrine carcinoma, neuroendocrine breast carcinoma, neuroendocrine carcinoma, tumor

## Abstract

**Background:**

Primary neuroendocrine breast carcinomas (NEBCs) are an extremely rare and underrecognized subtype of mammalian carcinoma. The prognostic factors for NEBCs remain controversial.

**Methods:**

In this multicenter retrospective study, the prognostic factors for patients with primary NEBCs who underwent surgery and had a pathologically confirmed diagnosis of neuroendocrine carcinoma in China and the United States were examined. The endpoints were disease‐free survival (DFS) and overall survival (OS).

**Results:**

A total of 51 Chinese patients and 98 US patients were included. In the Chinese cohort, tumor grade and Ki‐67 levels were prognostic factors for DFS in univariate analysis (hazard ratio [HR] = 5.11 [1.67–15.60], *p* = 0.004; HR = 57.70 [6.36–523.40], *p* < 0.001, respectively) and multivariate analysis (HR = 100.52 [1.33–7570.21], *p* = 0.037; HR = 31.47 [1.05–945.82], *p* = 0.047, respectively). In the US cohort, age was an important prognostic factor for OS in univariate analysis (HR = 1.09 [1.04–1.15], *p* = 0.001). The random effects model for the combined cohorts revealed age and positive expression of estrogen receptor (ER) as potential prognostic factors for OS (HR = 1.08 [1.01–1.14], *p* = 0.015; HR = 0.10 [0.02–0.44], *p* = 0.003, respectively).

**Conclusions:**

Tumor grade and Ki‐67 levels are important prognostic factors for DFS of patients with primary NEBCs. Age and ER status are important prognostic factors for OS of patients with primary NEBCs.

## Graphical abstract

Tumor grade and Ki‐67 levels are important prognostic factors for DFS of patients with primary neuroendocrine breast carcinomas, Ki‐67 > 55% had a worse prognosis than <55%.

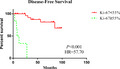

## 1 INTRODUCTION

Neuroendocrine neoplasms (NENs) are a relatively rare and heterogeneous group of tumors originating from the neuroendocrine system.^1^ Studies have demonstrated the importance of tumor classification, grading, and staging when assessing the prognosis of patients with NENs. However, the prognostic factors for NENs are not well‐known. There was a significant increase in the reported annual age‐adjusted incidence of neuroendocrine tumors (NETs) from 1973 (1.09/100,000) to 2004 (5.25/100,000) in the Surveillance, Epidemiology, and End Results (SEER) database. Therefore, it has become increasingly important to study the prognostic factors for patients with NENs.^2^, ^3^, ^4^

Primary neuroendocrine breast carcinomas (NEBCs) are a very rare group of tumors^5^ and account for <1% of all NETs and <0.1% of invasive breast cancers.^6^, ^7^ The neuroendocrine differentiation of breast cancer was first proposed in 1963.^8^ In 2003, the World Health Organization acknowledged it as a distinct solid tumor in the *Pathology and Genetics of Tumors of the Breast and Female Genital Organs*, with a diagnostic criterion of >50% of tumor cells expressing neuroendocrine markers.^9^

Some studies have shown that NEBCs have a poor prognosis.^6^, ^10^, ^11^, ^12^, ^13^ Conversely, a retrospective study of 224 patients with NETs showed that breast carcinomas that expressed high levels of neuroendocrine markers and cytomorphologic features were associated with a better prognosis.^14^ Considering the contradictory nature of these findings, the prognostic factors that affect NEBCs remain controversial and require further study. In addition, according to the 2012 WHO classification, primary NEBCs accounted for 2%–5% of breast cancers^15^; due to the low incidence of NEBCs despite the increasing numbers, little is known on their optimal management. Owing to the scarcity of reported cases of NEBCs, we herein used Chinese and US cohorts to reveal the prognostic factors for NEBCs.

## 2 METHODS

### 2.1 Chinese cohort: Patients and variables

Data of patients who visited the Cancer Hospital Chinese Academy of Medical Sciences and Beijing Cancer Hospital between 2000 and 2018 were retrospectively collected. Patients with a pathologically confirmed diagnosis of neuroendocrine carcinoma and those with complete pathological information were included. Exclusion criteria of patients were as follows^1^: no surgical treatment^2^; primary diagnosis of metastatic tumor; and^3^ missing survival information. The pathological diagnosis of all patients was confirmed by consultation with a physician from the Department of Pathology, Cancer Hospital Chinese Academy of Medical Sciences. All patients provided written informed consent.

Data on tumor size, number of positive lymph nodes, the 7th American Joint Committee on Cancer (AJCC) tumor stage, tumor grade, estrogen receptor (ER) status, progesterone receptor (PR) status, human epidermal growth factor receptor 2 (HER2) status, chromogranin A (CgA), and synaptophysin (Syn) positivity were retrospectively collected from pathology reports. ER and PR positivity were defined as any positive nuclear staining (i.e., ≥1%). HER2 positivity was defined as immunohistochemistry (+++) or immunohistochemistry (++) with gene amplification confirmed by fluorescence in situ hybridization. Information on the treatment of patients was obtained from follow‐up data. Information on the survival status of patients was obtained by telephone follow‐up. In this study, disease‐free survival (DFS) was defined as the time since radical surgery to disease recurrence, metastasis, the last follow‐up, or patient death. In addition, overall survival (OS) was defined as the time since diagnosis to death from any cause or the last follow‐up.

Additional patients were included who visited Beijing Hospital between 2008 and 2017. Information from the medical records of these patients is publicly available.^16^

### 2.2 US cohort: Patients and variables

The SEER database was searched for all NEBCs that were diagnosed between 2010 and 2015 because the registration of the HER2 status in the SEER database commenced in 2010. The following inclusion criteria were used for data screening^1^: female breast cancer had to be the first and only cancer diagnosis^2^; the histology type of the patients had to be infiltrating carcinoma (International Classification of Disease, 3rd edition, ICD‐O‐3 code 8013/3, 8246/3, and 8574/3); and^3^ patients should have undergone mastectomy or lumpectomy. Patients with unknown diagnosis dates and unknown survival times were excluded from this study.

The following variables were extracted from the selected SEER database: age at diagnosis, race or ethnicity, laterality (right or left side), tumor grade (well‐differentiated, moderately differentiated, or poorly differentiated), the 7th AJCC tumor stage, ER status, PR status, HER2 status, and history of undergoing radiotherapy or chemotherapy. The OS and cause‐specific survival were used as endpoints.

### 2.3 Statistics

The cutoff for Ki‐67 levels was determined using X tile 3.6.1 software (Yale University, USA). Categorical information was tested using a chi‐squared test or Fisher's exact test. The Wilcoxon rank sum test was used for ordered data. A Cox regression model was used for survival analysis. Variables with a *p*‐value <0.25 in univariate analysis were selected as independent variables and analyzed by multivariate Cox regression. A random effects model (REM) was used to combine hazard ratios (HRs). All analyses were performed in R 4.0.1. Survival curves were plotted using GraphPad Prism 6. All were tested using a two‐sided test. A *p‐*value <0.05 was considered statistically significant.

## 3 RESULTS

### 3.1 Clinical characteristics of the cohorts in China and the United States

A total of 51 Chinese patients were included in the study: 17 from the Cancer Hospital Chinese Academy of Medical Sciences, 27 from Beijing Hospital, and 7 from the Beijing Cancer Hospital. The mean age (range) of all patients was 62.29 years (24–92 years) with a standard deviation of 15.45 years. Clinical characteristics of the cohorts are shown in Table 1. Most patients were in the early stage of tumor growth without lymph node metastasis (N0). According to the AJCC staging system, 13 (27.08%) and 32 (66.67%) patients were in stage I and II, respectively. In addition, according to the degree of differentiation, most patients showed highly or moderately differentiated tumors (*n* = 35, 71.47%). Most patients were ER/PR‐positive and HER2‐negative. For treatment, all patients underwent surgery, and most patients received chemotherapy (*n* = 31, 60.78%) and endocrine therapy (*n* = 37, 72.55%) but not radiotherapy (*n* = 40, 78.43%).

**TABLE 1:** Clinical characteristics and treatment strategies of patients in China and the United States

	China		US		*p‐*value
	*N*	%	*N*	%	
Overall	51		98		
Laterality					0.036
Left	32	64.00	52	53.06	
Right	16	32.00	46	46.94	
Bilateral	2	4.00	0	0	
Unknown	1		0		
pT stage					0.186
T1	19	37.25	32	32.99	
T2	31	60.78	51	52.58	
T3	0	0	11	11.34	
T4	1	1.96	3	3.09	
Unknown			1		
pN stage					0.620
N0	34	70.83	66	67.35	
N1	12	25.00	25	25.51	
N2	1	2.08	5	5.10	
N3	1	2.08	2	2.04	
Unknown	3				
AJCC 7th Stage					0.992
I	13	27.08	30	30.93	
II	32	66.67	56	57.73	
III	3	6.25	11	11.34	
Unknown	3		2		
Tumor grade					0.005
1 and 2	35	71.43	43	46.24	
3	14	28.57	50	53.76	
Unknown	2		5		
ER					0.668
Positive	42	82.35	74	78.72	
Negative	9	17.65	20	21.28	
Unknown	0		4		
PR					0.550
Positive	40	78.43	68	72.34	
Negative	11	21.57	26	27.66	
Unknown	0		4		
HER2					0.049
Positive	5	12.82	3	3.23	
Negative	34	87.18	90	96.77	
Unknown	12		5		
Surgery					<0.001
Mastectomy	39	88.64	38	38.78	
Lumpectomy	5	11.36	60	61.22	
Unknown	7				
Radiotherapy					<0.001
Yes	11	21.57	53	54.08	
No	40	78.43	45	45.92	
Chemotherapy					0.167
Yes	31	60.78	47	47.96	
No	20	39.22	51	52.04	
Ki‐67 levels					
<55%	36	85.71			
≥55%	6	14.29			
Unknown	9				
CgA					
Positive	31	81.58			
Negative	7	18.42			
Unknown	13				
Syn					
Positive	28	80.00			
Negative	7	20.00			
Unknown	16				
Endocrine therapy					
Yes	37	72.55			
No	14	27.45			
Race/Ethnicity					
White			85	87.63	
Black			8	8.25	
Asian or Pacific Islander			4	4.12	
Unknown			1		

Abbreviations: AJCC, American Joint Committee on Cancer; CgA, chromogranin A; ER, estrogen receptor; HER2, human epidermal growth factor receptor 2; PR, progesterone receptor; Syn, synaptophysin.

In addition, 98 US patients were included from the SEER database; these patients had a mean age (range) of 63.5 years (34–92 years) and a standard deviation of 15.98 years. Similar to the Chinese patients, most of these patients were in the early stage of tumor growth. The majority of the patients were in the T2 stage (*n* = 51, 52.58%) and did not have lymph node metastasis (N0; *n* = 66, 67.35%). However, the proportion of high‐pathological‐grade, poorly differentiated tumors was higher in the US patients (*n* = 50; 53.76%) than in the Chinese patients (*n* = 14; 28.57%). The majority of patients were ER/PR‐positive and HER2‐negative. Regarding the treatment, more patients underwent lumpectomy (60, 61.22%) than mastectomy (38, 38.78%). In contrast to Chinese patients, more US patients received radiotherapy (*n* = 53; 54.08% vs. 21.57%, *p* < 0.001).

### 3.2 Univariate and multivariate survival analysis of DFS in Chinese patients

**FIGURE 1:**
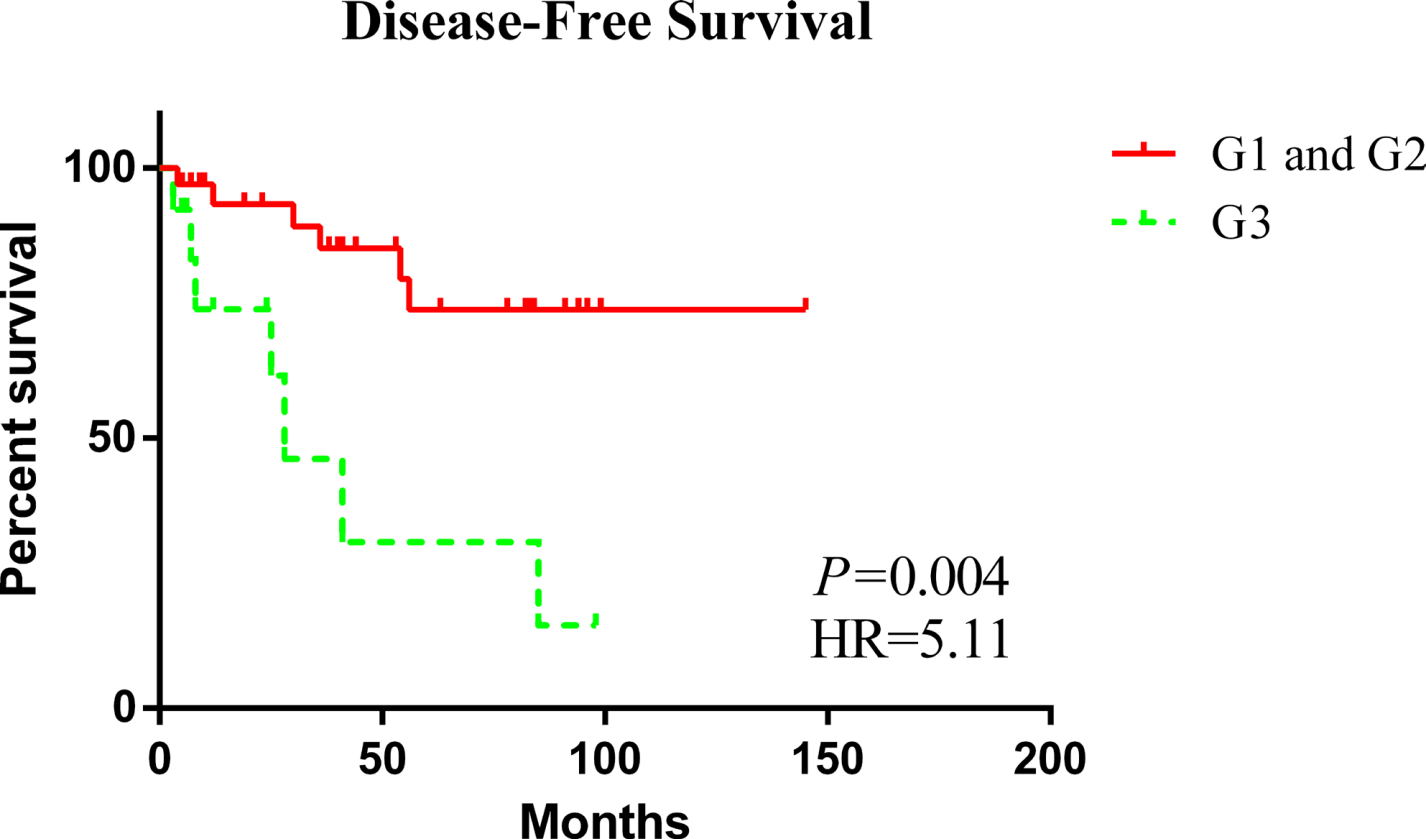
Kaplan–Meier curves of DFS according to tumor grades

**FIGURE 2:**
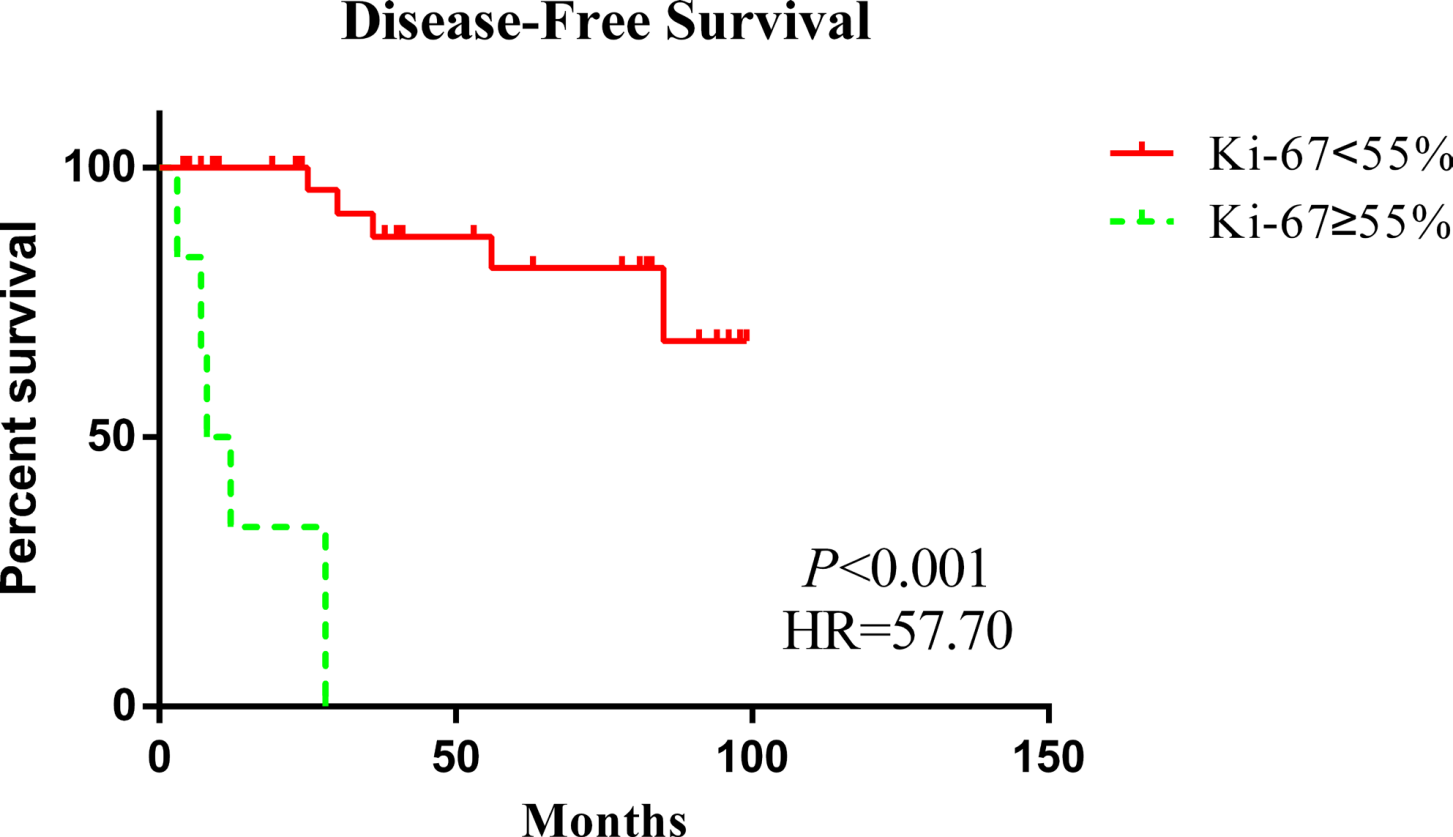
Kaplan–Meier curves of DFS according to Ki‐67 levels

The analysis of prognostic factors for DFS is shown in Table 2. Univariate Cox regression models showed that poorly differentiated tumors were associated with a worse prognosis than well‐differentiated and moderately differentiated tumors (HR = 5.11 [1.67–15.60], *p* = 0.004), Figure 1]. In addition, higher Ki‐67 levels were associated with a worse prognosis (HR = 57.70 [6.36–523.40], *p* < 0.001), Figure 2]. Owing to the lack of standardization and the variability in the cutoff points used to define a high Ki‐67 index of NEBCs, we analyzed the influence of different Ki‐67 cutoff levels (15%, 20%, 30%, and 55%) on the DFS of patients in China. We found that there were differences at the cutoff level of 55% both in univariate [HR = 57.70 (6.36–523.40), *p* < 0.001] and multivariate [HR = 31.47 (1.05–945.82), *p* < 0.05] analyses; the results are shown in Table S1. Furthermore, there could be an association between radiotherapy and DFS [HR = 2.80 (0.90–8.70), *p* = 0.074]. Age, stage, other pathological characteristics (ER, PR, HER2, and CgA), and other treatment strategies (e.g., endocrine therapy and chemotherapy) of patients did not show statistically significant differences.

**TABLE 2:** DFS of patients in China

	Univariate Cox regression model		Multivariate Cox regression model	
	HR (95% CI)	*p‐*value	HR (95% CI)	*p‐*value
Age	0.98 (0.94–1.01)	0.147	0.88 (0.78–1.00)	0.052
AJCC 7th stage	1.19 (0.40–3.50)	0.756		
Tumor grade	5.11 (1.67–15.60)	0.004	100.52 (1.33–7570.21)	0.037
N stage	0.92 (0.28–3.04)	0.891		
ER	0.39 (0.11–1.46)	0.162	0.51 (0.03–8.94)	0.648
PR	1.47 (0.32–6.64)	0.618		
HER2	2.29 (0.49–10.64)	0.290		
Ki‐67 levels	57.70 (6.36–523.40)	<0.001	31.47 (1.05–945.82)	0.047
CgA	0.40 (0.10–1.58)	0.187	0.04 (0.001–1.12)	0.058
Surgery	0.71 (0.16–3.22)	0.658		
Radiotherapy	2.80 (0.90–8.70)	0.074	0.04 (0.001–1.77)	0.096
Endocrine therapy	0.72 (0.22–2.36)	0.588		
Chemotherapy	1.52 (0.47–4.95)	0.486		

Abbreviations: HR, hazard ratio; AJCC, American Joint Committee on Cancer; CgA, chromogranin; CI, confidence interval; ER, estrogen receptor; HER2, human epidermal growth factor receptor 2; PR, progesterone receptor.

With adjustment for covariates, multivariate Cox regression analysis showed an association between worse prognosis with a higher tumor grade (HR = 100.52 [1.33–7570.21], *p* = 0.037) and higher Ki‐67 levels (HR = 31.47 [1.05–945.82], *p* = 0.047).

### 3.3 Survival analysis of overall survival in Chinese and US cohorts

Univariate Cox regression analysis of OS of Chinese and US patients is given in Table 3. Age, pathological characteristics, and treatment strategies did not have a statistically significant impact on OS of Chinese patients. In the US patients, increasing age had an adverse effect on prognosis (HR = 1.09 [1.04–1.15], *p* = 0.001). After adjustment, a multivariate Cox regression analysis was performed for Chinese and US patients, as shown in Table 4. HRs were combined using a REM. Consequently, age (HR = 1.08 [1.01–1.14], *p* = 0.015) and ER positivity (HR = 0.10 [0.02–0.44], *p* = 0.003) predicted a statistically significantly better prognosis. The impact of N stage and chemotherapy on prognosis needs to be determined.

**TABLE 3:** Univariate Cox regression model of OS analysis for patients in China and the United States

	HR (95% CI)	*p‐*value
China		
Age	1.02 (0.97–1.10)	0.362
AJCC 7th stage	0.50 (0.10–2.38)	0.380
Tumor grade	1.11 (0.12–10.10)	0.924
N stage	0.57 (0.06–5.13)	0.616
ER	0.37 (0.04–3.50)	0.382
HER2	1.84 (0.20–16.56)	0.588
CgA	0.53 (0.05–5.16)	0.582
Surgery	0.40 (0.04–3.55)	0.408
Endocrine therapy	1.32 (0.15–11.88)	0.804
Chemotherapy	0.17 (0.02–1.56)	0.119
US		
Age	1.09 (1.04–1.15)	0.001
N stage	0.34 (0.08–1.55)	0.165
AJCC 7th stage	1.26 (0.56–2.87)	0.576
Tumor grade	2.42 (0.31–18.70)	0.396
Race		
White	1 (reference)	
Black	0.82 (0.11–6.32)	0.848
Asian or Pacific islander	1.92 (0.25–14.81)	0.533
ER	0.39 (0.11–1.38)	0.144
PR	0.51 (0.14–1.84)	0.306
Surgery	1.34 (0.45–3.99)	0.601
Radiotherapy	0.65 (0.22–1.95)	0.447
Chemotherapy	0.94 (0.31–2.78)	0.908

Abbreviations: AJCC, American Joint Committee on Cancer; CgA, chromogranin A; CI, confidence interval; ER, estrogen receptor; HER2, human epidermal growth factor receptor 2; HR, hazard ratio; PR, progesterone receptor.

**TABLE 4:** Multivariate Cox regression model of OS analysis for patients in China and the United States

	HR (95% CI)	SEM	*p‐*value
Age			
China	1.03 (0.94–1.13)	0.05	0.486
US	1.10 (1.03–1.17)	0.03	0.003
REM	1.08 (1.01–1.14)	0.03	0.015
N stage			
China	1.18 (0.11–12.47)	1.20	0.888
US	0.55 (0.11–2.77)	0.83	0.466
REM	0.70 (0.18–2.68)	0.68	0.607
ER			
China	0.04 (0.001–1.76)	0.94	0.096
US	0.19 (0.05–0.80)	0.72	0.022
REM	0.10 (0.02–0.44)	0.77	0.003
Chemotherapy			
China	0.07 (0.004–1.26)	1.48	0.070
US	2.44 (0.65–9.11)	0.67	0.186
REM	0.53 (0.02–16.58)	1.76	0.717

Abbreviations: CI, confidence interval; ER, estrogen receptor; HR, hazard ratio; REM, random effects model; SEM, standard error of mean.

## 4 DISCUSSION

Primary neuroendocrine carcinomas of the breast are extremely rare and therefore controversial in terms of diagnosis, treatment, and prognosis. This study was a multicenter retrospective study and included population data from the SEER database. A total of 149 patients were included to analyze the prognostic factors in patients with NEBCs.

The mean age of the patients was 62.56 years, with most being postmenopausal. Survival analysis of the SEER database population and the combined analysis of Chinese and the US cohorts suggested that age was a significant factor that affected OS (HR = 1.09 [1.04–1.15], *p* = 0.001, HR = 1.08 [1.01–1.14], *p* = 0.015, respectively). Several previous studies have reported age as an important prognostic factor for OS.^17^, ^18^ Zhou et al. reported that age at diagnosis significantly affects OS of patients with pancreatic NETs (HR = 1.92 [1.69–2.17], *p* < 0.001). A multivariate Cox regression analysis of breast cancer patients by Wray et al. showed that age was an independent predictor for OS (HR = 1.04 [1.04–1.05], *p* < 0.001).

There were differences in the nuclear grades of tumors in the Chinese and US cohorts. Chinese patients showed a greater tendency to have well‐differentiated and moderately differentiated tumors, whereas their US counterparts predominantly showed poorly differentiated tumors. For Chinese patients, poor differentiation had a negative effect on DFS (HR = 100.52 [1.33–7570.21], *p* = 0.037). Wei et al.’s prognostic study of NEBCs showed that among patients with NETs, those with a higher tumor grade tended to have shorter DFS and OS (*p* = 0.005 and 0.003, respectively). Li et al. analyzed the prognostic factors in patients with pancreatic NETs and showed that G3 negatively impacted prognosis more strongly than G1 (HR = 4.067 [2.952–5.603], *p* < 0.001).^12^, ^19^ This may be related to the biological behavior of the tumor. Poorly differentiated tumor cells are more likely to metastasize. In addition, compared to ER(−) patients, ER(+) patients had a better prognosis (HR = 0.10 [0.02–0.44], *p* = 0.003), which may be attributed to them receiving endocrine therapy. In Chinese patients, Ki‐67 ≥ 55% was associated with a worse prognosis than Ki‐67 < 55% (HR = 31.47 [1.05–945.82], *p* = 0.047). Though lack of standardization and variability in breast cancer,^20^ the St. Gallen consensus 2009 proposed three categories: low (<15%), intermediate (16%–30%), and high (>30%).^21^ Gallen (2013) changed the cutoff point to 20% with the option to use local laboratory values.^22^ Besides, most Chinese experts agree that Ki‐67 < 15% indicates low expression and >30% indicates high expression.^23^ However, we found that there were differences at the cutoff level of 55% both in univariate (HR = 57.70 [6.36–523.4], *p* < 0.001) and multivariate (HR = 31.47 [1.05–945.82], *p* < 0.05) analyses and that multivariate analyses were negative if the Ki‐67 cutoff level was 15%, 20%, or 30%. In a report, 55% Ki‐67 was defined as the threshold that could change the tumor response to platinum/etoposide chemotherapy.^24^ Chen et al. reported that Ki‐67 levels were higher in patients with postoperative recurrent disease within 5 years than in patients without recurrence (area under the curve [AUC] = 0.860 [0.805–0.916]).^25^ This was because Ki‐67 could induce tumor proliferation. According to the multivariate Cox regression analysis of DFS in Chinese patients, CgA may be associated with a better prognosis (HR = 0.04 [0.001–1.12], *p* < 0.1), which is consistent with the results for gastroenteropancreatic neoplasms.^26^

There was a difference in the surgical approach used for Chinese and US patients. The Chinese patients were more likely to receive mastectomies, whereas the US patients were more likely to receive lumpectomies. This may be related to cultural differences.^27^, ^28^ In addition, Chinese women have relatively small breasts and may not always be eligible for breast‐conserving surgery. The proportion of patients with NEBCs treated with radiation therapy was lower in China than in the US. Breast‐conserving surgery with radiation therapy is a widely accepted standard method and allows breast preservation in most patients with early‐stage breast cancer. More patients are treated with breast‐conserving therapy in the United States than in China, and therefore, more patients in the US receive radiation therapy. According to a univariate Cox regression analysis of DFS in Chinese patients, radiotherapy may be associated with a poor prognosis (HR = 2.8 [0.90–8.70], *p* < 0.1). However, patients with breast NETs treated with radiotherapy reportedly had longer median OS (156 vs. 88 months) and median DFS (138 vs. 80 months) than those who did not receive radiotherapy.^12^

The use of chemotherapy for NEBCs remains controversial. NEBCs are treated according to the ER and HER2 statuses of the patient.^5^ Neuroendocrine components can be controlled by anthracyclines.^27^ Patients treated with chemotherapy reportedly had a DFS of ≤36 months.^28^ However, patients who received chemotherapy had shorter OS and DFS than those who did not.^12^ Many studies have shown that NETs often exhibit overexpression of growth inhibitors.^29^, ^30^, ^31^ Currently, growth inhibitory analogs (mainly somatostatin analogs [SSAs]) are used as the first‐line treatment or adjuvant therapy after surgery for well‐differentiated gastroenteropancreatic NETs.^32^ A meta‐analysis by Dolan et al. showed that SSAs showed good results in the treatment of metastatic breast cancer with few adverse effects.^33^ However, to our knowledge, no treatment that uses SSAs have been reported for breast NETs. In addition, there are case reports of good outcomes with peptide receptor radionuclide therapy in breast cancer patients with neuroendocrine differentiation.^34^ Therefore, the role of pharmacotherapy in NEBCs requires further study.

There are several potential limitations of this study. First, the SEER database has deficiencies in data related to endocrine therapy and the recurrence or metastasis status. This means that it is difficult to assess the role of endocrine therapy in the US population and perform DFS analyses. Second, because NEBCs are extremely rare, it is difficult to expand the sample size as all six patients with Ki‐67 ≥ 55% died, which made OS analysis impossible. Finally, though menstrual status and BMI are important factors of breast cancer, there are no available data for these factors in the SEER database. Besides, as the data of Chinese cohort were retrospective and from multiple centers, it was difficult to collect menstrual status and BMI data for analysis. However, because screening techniques continue to improve and the number of patients identified as having NEBCs continues to increase, the best treatment modalities and treatment options for this rare tumor will continue to be explored.

## 5 CONCLUSION

Tumor grade and Ki‐67 levels are important prognostic factors for DFS of primary neuroendocrine carcinomas of the breast. CgA and radiotherapy are potential prognostic factors for DFS. Age and ER status are important prognostic factors for the OS of primary neuroendocrine carcinomas of the breast.

## AUTHOR CONTRIBUTIONS

Shu‐tao Ma and Ding‐yuan Wang designed the study, performed most of the investigations, and data analysis. Yi‐bing Liu and Hui‐jing Tan contributed significantly to analysis and manuscript preparation. Yue‐yue Ge provided data and wrote the manuscript. Yihebali Chi and Bai‐lin Zhang reviewed and edited the manuscript. All authors read and approved the manuscript.

## ETHICS STATEMENT

The protocol was approved by the Institutional Review Board at each participating institution center and complied with good clinical practice guidelines, as well as the Declaration of Helsinki.

## Supporting information


Table S1

## Data Availability

The datasets generated and analyzed during the present study are available from the corresponding author on reasonable request.
