# Supplementary material for: Prognostic factors of primary neuroendocrine breast cancer: A population‐based study
Source: Cancer Med. 2022 May 2;11(13):2533–40. doi: 10.1002/cam4.4557 (PMC9249978; doi:10.1002/cam4.4557)
Supplement: Supplementary file 1 — Table S1 [file CAM4-11-2533-s001.docx]

Supplementary Table 1. The influence of different Ki-67 cut-off levels on DFS of patients in China

|  |  | Univariate Cox regression model | | Multivariate Cox regression model | |
| --- | --- | --- | --- | --- | --- |
| Ki-67 levels | N | HR (95% CI) | *p*-value | HR (95% CI) | *p*-value |
| 15% |  |  |  |  |  |
| <15% | 14 | 1 (reference) |  | 1 (reference) |  |
| ≥15% | 28 | 2.77 (0.58–13.15) | 0.199 | 9.76 (0.37–254.70) | 0.171 |
| 20% |  |  |  |  |  |
| <20% | 16 | 1 (reference) |  | 1 (reference) |  |
| ≥20% | 26 | 3.28 (0.69–15.49) | 0.134 | 12.75 (0.56–291.17) | 0.111 |
| 30% |  |  |  |  |  |
| <30% | 24 | 1 (reference) |  | 1 (reference) |  |
| ≥30% | 18 | 4.61 (1.17–18.14) | 0.029 | 9.97 (0.40–250.16) | 0.162 |
| 55% |  |  |  |  |  |
| <55% | 36 | 1 (reference) |  | 1 (reference) |  |
| ≥55% | 6 | 57.70 (6.36–523.40) | <0.001 | 31.47 (1.05–945.82) | 0.047 |

Note: HR, hazard ratio; CI, confidence interval
